# Supplementary figures and images for: Renal interstitial cells promote nephron regeneration by secreting prostaglandin E2
Source: eLife. 2023 Jan 16;12:e81438. doi: 10.7554/eLife.81438 (PMC9943066; doi:10.7554/eLife.81438)

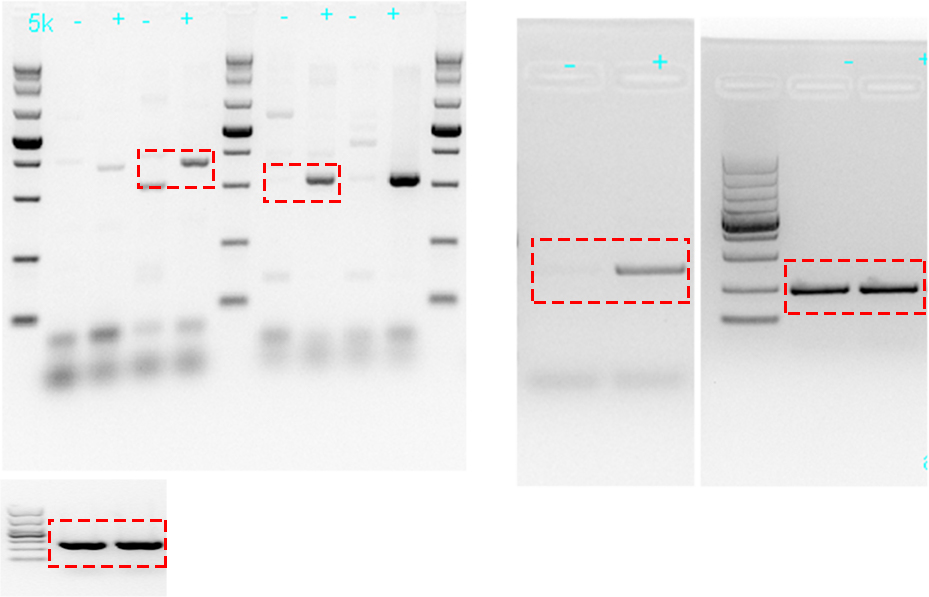

Supplement: Figure 2—source data 1. [file elife-81438-fig2-data1.zip › Figure 2-source data 1 marked.jpg]

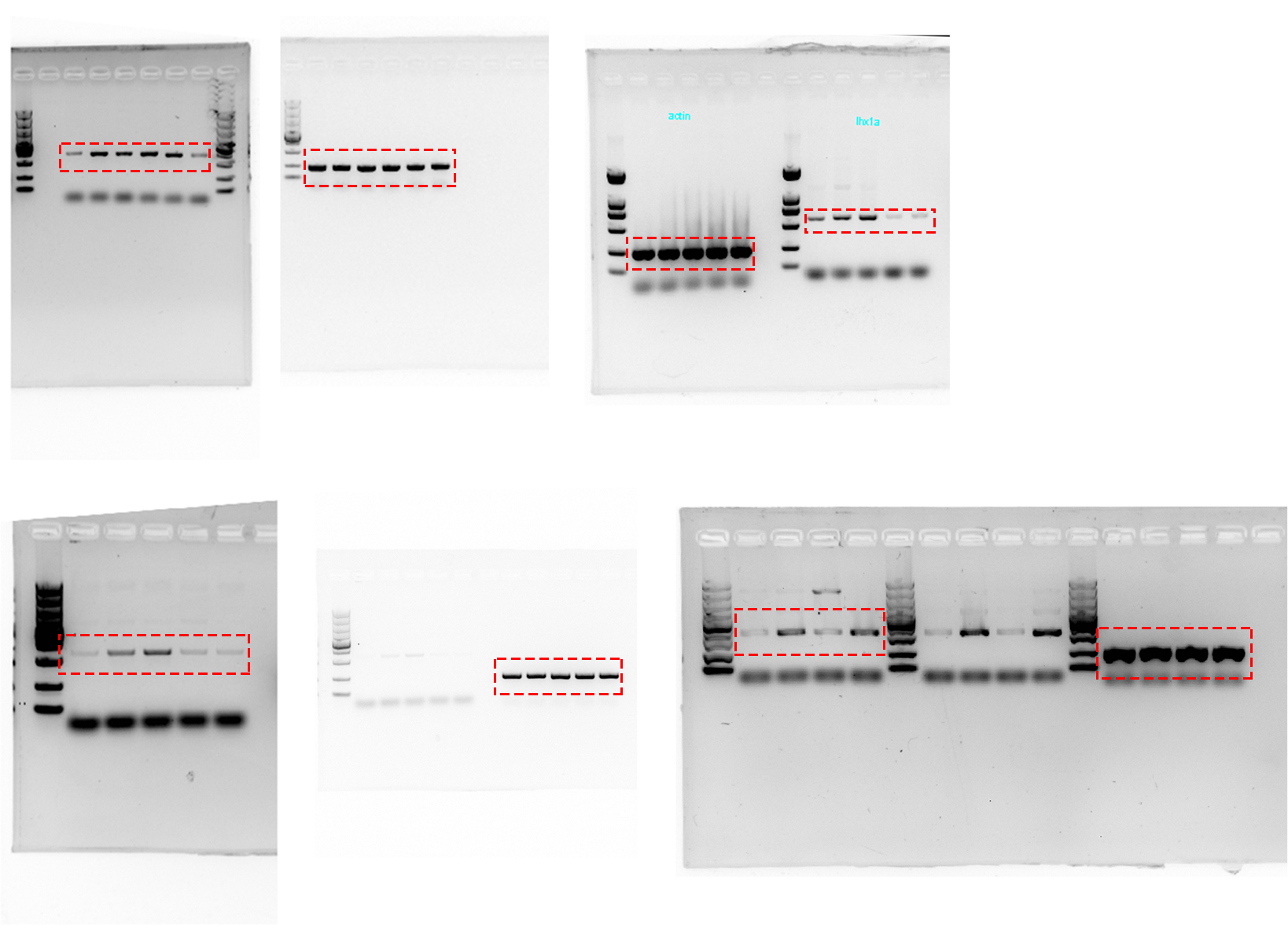

Supplement: Figure 3—source data 2. [file elife-81438-fig3-data2.zip › Figure 3-source data 2 marked.jpg]

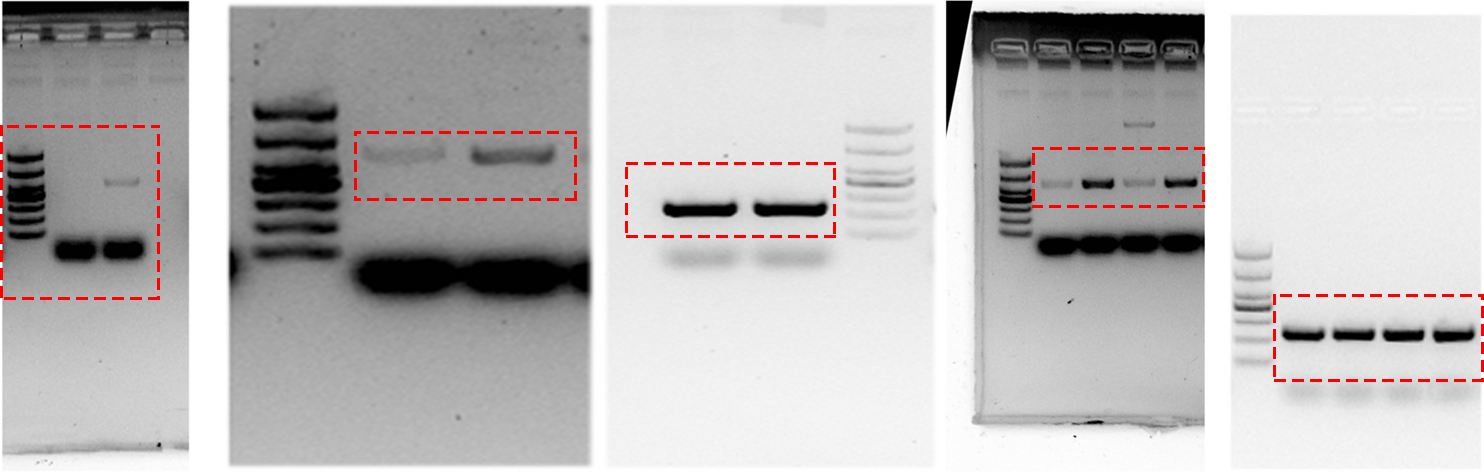

Supplement: Figure 5—source data 1. [file elife-81438-fig5-data1.zip › Figure 5-source data 1 marked.jpg]

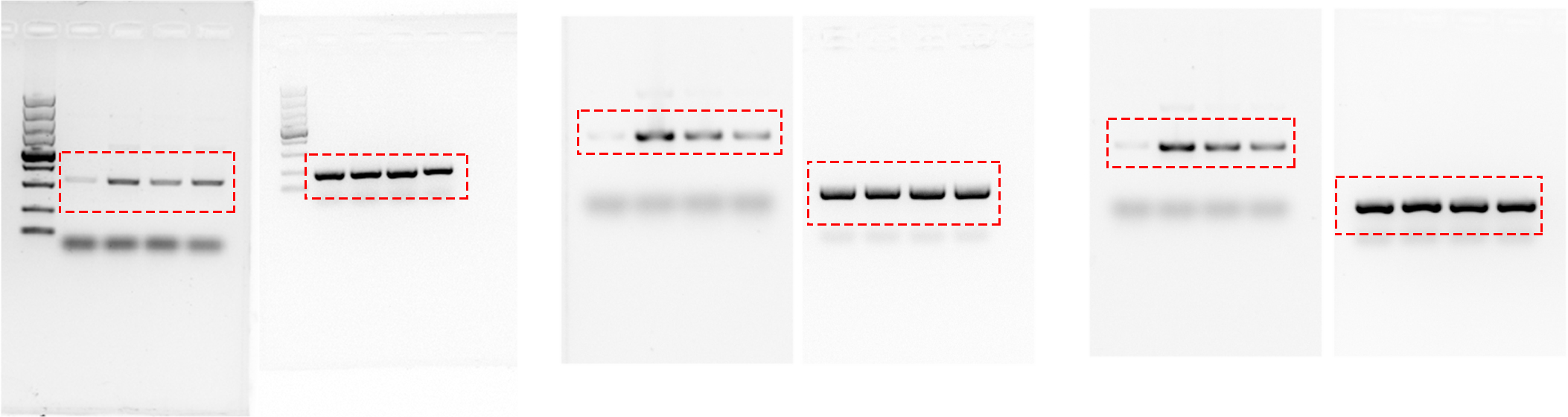

Supplement: Figure 6—source data 1. [file elife-81438-fig6-data1.zip › Figure 6-source data 1 marked.jpg]

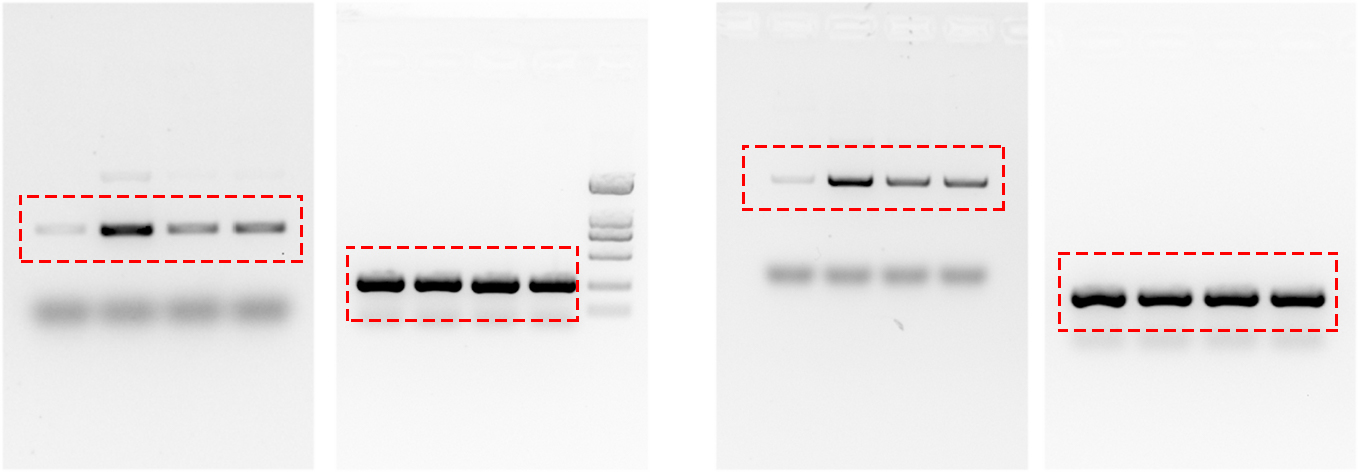

Supplement: Figure 7—source data 1. [file elife-81438-fig7-data1.zip › Figure 7-source data 1 marked.jpg]
